# A Video-Based Intervention Supporting Spanish-Speaking Fathers of Infants in the Neonatal Intensive Care Unit: Open-Label Pilot Study

**DOI:** 10.2196/72520

**Published:** 2025-12-09

**Authors:** Janine Bernardo, Elise Belkin, Suzanne Hally, Sara Boyle, Eric Van, Jose Tascon-Arcila, Emily Herzberg, Juan Matute, Paul Lerou

**Affiliations:** 1 Division of Newborn Medicine, Department of Pediatrics, Massachusetts General Hospital, 55 Fruit Street, BUL 2, Boston, MA, 02114, United States, 1 6177249040; 2 Department of Pediatrics, Boston Children's Hospital, Boston, United States; 3 Division of Neonatology, Department of Pediatrics, Perelman School of Medicine at the University of Pennsylvania, Children's Hospital of Philadelphia, Philadelphia, United States

**Keywords:** neonatal intensive care unit, NICU, language other than English, Spanish-speaking, fathers, infants, videos, pilot study, feasibility, accessibility

## Abstract

This open-label pilot study showed the feasibility and acceptability of an educational video intervention for Spanish-speaking fathers in the neonatal intensive care unit to increase knowledge and confidence, decrease anxiety, and improve parenting competency.

## Introduction

Parental involvement for infants admitted to the neonatal intensive care unit (NICU) encapsulates the earliest, critical moments of interaction with a child, yet can be accompanied with stress, anxiety, and uncertainty [1]. Parents who speak languages other than English are at increased risk of disrupted communication, stress, and suboptimal care [2]. NICU fathers who are from minority racial and ethnic groups have higher rates of depression and anxiety than fathers who are not [3,4], in part due to ineffective communication and targeted support/resources, resulting in feeling undervalued [3,5,6].

Various interventions have been implemented to support the well-being and involvement of NICU fathers including psychosocial support [7], written materials [8], enhanced nursing support [9], narrative writing [10], skin-to-skin, and increased physical interaction [11,12]. Despite being inexpensive and distributable, there is a paucity of video-based interventions [12] targeting Spanish-speaking NICU fathers. Prior work demonstrates that NICU fathers find staff education, particularly from nurses, pivotal in overcoming low knowledge and uncertainty, leading to improved confidence and overall NICU experience [13]. Thus, a video-based, educational intervention for Spanish-speaking fathers would provide an opportunity to better support fathers and the child-father relationship at its earliest stages.

The Bronfenbrenner’s ecological systems theory posits an individual’s health and development is shaped by varying layers of environmental and social relations from the micro to macro scale [14]. Over the course of a lifetime, an individual’s development evolves through shared, increasingly complex interactions between the individual and their environment. This project aimed to support the early child-father relationship to improve fathers’ knowledge and confidence such that the reciprocal, foundational nature of their relationship may flourish.

We performed a single-arm intervention feasibility study using short videos on high-yield NICU topics. Our objective was to assess whether the intervention “Los Padres de los Pajaritos” (“the fathers of little birds”) would be feasible and acceptable to Spanish-speaking NICU fathers to assess improved knowledge and confidence, decreased anxiety, and improved parenting competency to lay the groundwork for future, larger-scale efficacy trials.

## Methods

### Participants

The study population included Spanish-speaking and Spanish-reading fathers with infants admitted to a Level IV NICU or Level II special care nursery (SCN) at a single, urban center from July 1, 2023, to March 31, 2024. Of note, in 2021, 7% of NICU/SCN parents identified Spanish as their preferred language. Infants who were transferred were followed after transfer, as able. The sample size was 10 fathers to identify a mean difference of 2 with ±2 standard deviations for a 5-point pre/post scale.

### Video Intervention

Three short videos (5‐10 min each) were written, edited, and recorded by neonatologists, nurses, and lactation specialists on the following topics: NICU Orientation, Nursing Care, and Lactation/Breastfeeding Support. Videos were translated into Spanish by native Spanish-speaking physicians and recorded by qualified interpreters. Videos were filmed by a trained medical videographer.

NICU Orientation introduced NICU team members, toured the unit, explained rounds, and provided information on interpretive services. The Nursing Care video demonstrated bedside skills including changing diapers, taking temperatures, swaddling, and performing skin-to-skin. The Lactation/Breastfeeding Support video discussed the benefits of breast milk using Centers for Disease Control and Prevention–generated resources, support for lactating mothers, and cleaning pump parts [15]. Fathers of patients admitted directly to the SCN were only offered Nursing Care and Lactation/Breastfeeding Support videos.

### Recruitment and Data Collection

Eligible fathers were identified through electronic medical record notifications for mother’s language preference and confirmed with the medical team before being approached for enrollment (Table 1). Preintervention surveys were administered within a week of admission by a research assistant and included age, employment, education, relationship status, insurance type, country of origin, and neonatal descriptors. Videos were distributed by a research assistant who demonstrated the use of QR codes to access videos. Fathers were instructed to view all videos in a preferred location either on a hospital iPad or personal device on their own time. Postintervention surveys were administered after fathers watched the videos and prior to discharge home. Upon completion, fathers were given remuneration including a gift card, books, and toys. Surveys were collected on iPads or paper and data were stored in REDCap (Research Electronic Data Capture). Standard of care, provided to all study participants, included teaching by bedside nurses, therapists, and providers. Social workers were available in the case of emotional distress, of which there were none.

**Table 1.** Study respondent sociodemographics.

Characteristic	Values
Average age (years)	31
Employed, n (%)	12 (92)
Highest level of education, n (%)	
Never graduated high school	5 (38)
High school diploma or GED^a^	7 (54)
Graduated college	1 (8)
Trade/vocational school	0 (0)
Advanced degree	0 (0)
Relationship status, n (%)	
Not in a relationship	0 (0)
In a relationship but not living with partner	1 (8)
In a relationship and living with partner but not married	7 (54)
Married and living together	5 (38)
Hispanic origin, n (%)	
Central American	6 (46)
Mexican, Mexican American, Chicano	1 (8)
Dominican Republic	2 (15)
South American	4 (31)
Languages spoken at home, n (%)	
Spanish	10 (77)
English and Spanish	3 (23)
Number of children (N=12 respondents), n (%)	
One	8 (62)
Two	3 (23)
Three	1 (8)
Four or more	0 (0)
Admission to, n (%)	
NICU^b^	11 (85)
SCN^c^	2 (15)
Previous child in NICU/SCN	0 (0)
Gestational age of child in NICU/SCN, n (%)	
<28 weeks	3 (23)
29‐32 weeks	3 (23)
33‐37 weeks	1 (8)
37 weeks and above	6 (46)
Insurance of child, n (%)	
Private	0 (0)
Public	9 (69)
No insurance	1 (8)
Not sure	3 (23)
Average visitation to NICU per week, n (%)	
I do not visit	0 (0)
1‐2 times a week	2 (15)
2‐5 times a week	3 (23)
More than 5 times a week	8 (62)
Transportation to the hospital, n (%)	
Drive	10 (77)
Public transit	1 (8)
Driver service (Uber, Lyft, taxi, etc)	2 (15)
Walk	0 (0)
I do not visit	0 (0)

a GED: general education development.

b NICU: neonatal intensive care unit.

c SCN: special care nursery.

### Ethical Considerations

This study was approved by the institutional review board of Mass General Brigham (number 2022P003021); all procedures were in accordance with the ethical standards of the institutional review board. Verbal, informed consent was obtained in Spanish and participants had an opportunity to ask questions in their preferred language. Confidentiality and privacy were strictly maintained. Participants were compensated with a US $30 gift card and age-appropriate toys and books.

### Measures

Feasibility was based on enrollment, with a goal of >70% to be considered good and >80% to be excellent [16]. Acceptability was defined as participant satisfaction with materials, with the above goals [17].

Preintervention surveys included demographics, a 5-point Likert scale on confidence/knowledge in infant care (1 being very low confidence/knowledge; 5 being very high), the Generalized Anxiety Disorder-7 (GAD-7) scale, and the Parenting Sense of Competence scale (PSOC). Postintervention surveys included 5-point Likert scales on confidence/knowledge in infant care, the GAD-7 scale, the PSOC, and feedback.

### Analysis

Demographic characteristics were described using frequencies and percentages. Preintervention and postintervention Likert scales, GAD-7 scores, and PSOC scores were analyzed using paired 1-sided *t* tests. An α level of 0.05 was used to signify statistical significance. Data were analyzed with IBM SPSS Statistics for Windows (version 28.0; IBM Corp).

## Results

**Figure 1.**
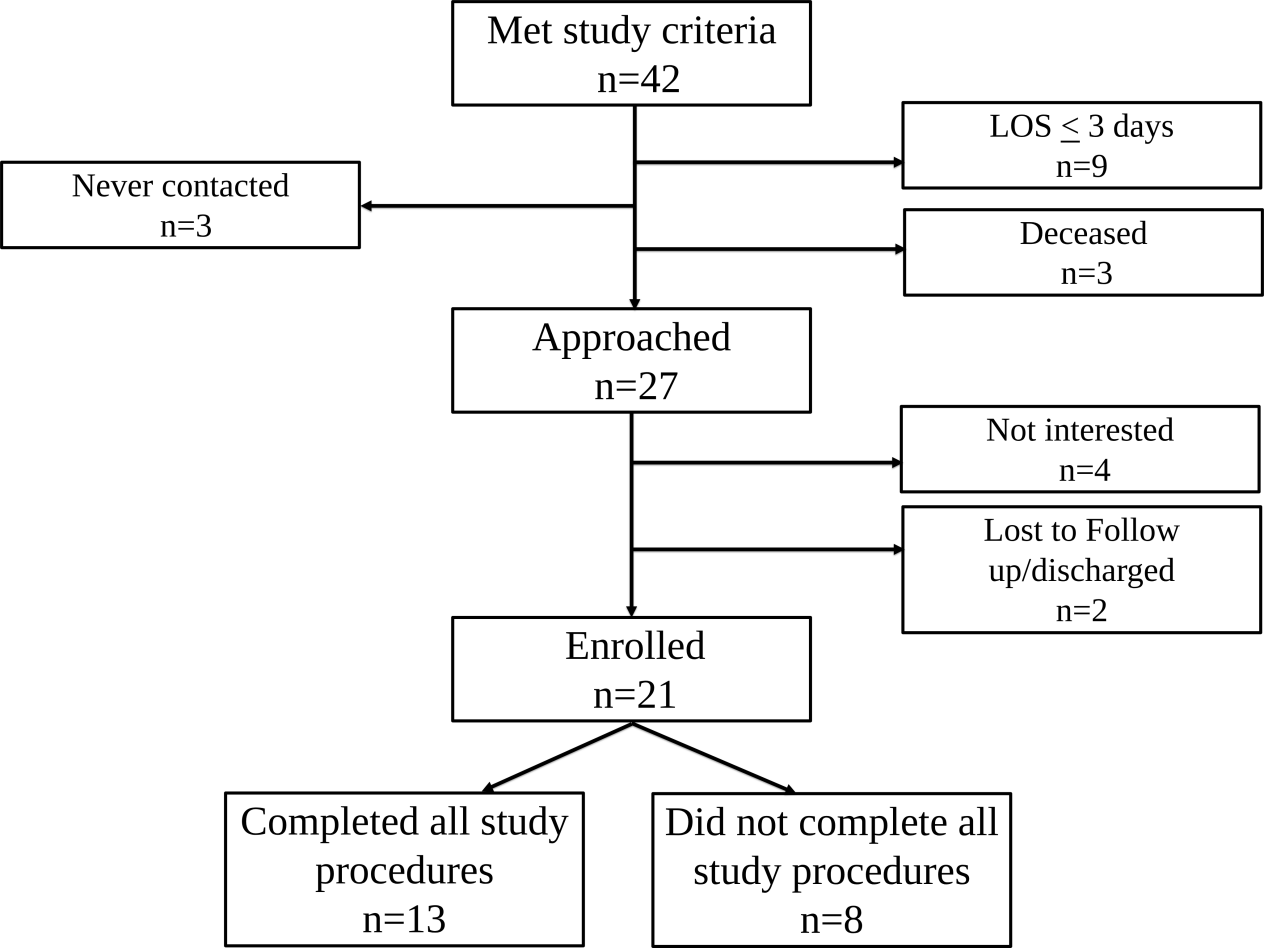
Schematic of eligibility, enrollment, and follow-up, showing the numbers of individuals who met study criteria, were approached and enrolled, and were lost to follow-up. Deceased refers to neonates who died during hospitalization. LOS: length of stay.

A total of 42 fathers met inclusion criteria; 27 [2] were approached and 21 (78%) were enrolled; 13 (62%) completed all study procedures (Figure 1). Demographics and clinical variables are reported in Table 1.

All fathers viewed at least 2 videos; 85% (11/13) viewed all videos. The Nursing Care video was most informative, while the Lactation Support video was the least watched. Likert scores on video feedback, including understandability, length, and quality, were all greater than 4.5 (Table 2). Confidence in caring for their infant showed a significant increase from 4.2 (95% CI 3.66-4.74) to 4.8 (95% CI 4.51-5.09)*, P*=.02, while knowledge increased (not significantly) from 4.09 (95% CI 3.46-4.72) to 4.69 (95% CI 4.29-4.97), *P*=.08. The average GAD-7 and PSOC scores did not change significantly (GAD-7: 4.75, 95% CI 2.56-6.92 and 3.33, 95% CI 1.49-5.17; *P*=.07; PSOC: 68.5, 95% CI 60.48-76.52 and 77.2, 95% CI 64.16-76.5; *P*=.31). Select comments were positive (Table 3). Qualitative video feedback included the following: “I found all the videos very interesting and educational,” “The videos speak very clearly and precisely...it is very important to know who you are dealing with and the trust that one places in them,” “I’m looking forward to seeing more videos,” and “Don’t stop sharing them because they are very important and you really do learn a lot.”

**Table 2.** Video usage descriptors*.* Self-reported video usage and feedback, suggestions for future interventions, and descriptions of timing of video completion are presented.

	Values
Video watch, n/N (%)	
Introduction to the NICU^a^	11/11 (100)
Orientation to nursing care	12/13 (92)
Lactation support	9/13 (69)
Most informative video, n/N (%)	
Introduction to the NICU	5/11 (45)
Orientation to nursing care	6/13 (46)
Lactation support	2/13 (15)
Least informative video, n/N (%)	
Introduction to the NICU	1/9 (11)
Orientation to nursing care	4/9 (44)
Lactation support	4/9 (44)
Suggested means of discussing care, n/N (%)	
Pamphlets/papers	5/13 (38)
Conferences	4/13 (31)
Bedside teaching	10/13 (77)
Other	
From other providers	1/13 (7)
Suggested topics for education	
Oxygen	—^b^
Reasons for babies to cry	—
Everything	—
Average days of viewing video following enrollment, n	4.08
Average days between viewing video and completing survey, n	7.79
Average days between completing preintervention and postintervention surveys, n	13.5

a NICU: neonatal intensive care unit.

b Not applicable.

**Table 3.** Video acceptability and qualitative feedback. Preintervention and postintervention data points are described, including knowledge and confidence Likert scales, Generalized Anxiety Disorder-7 scores, and Parenting Sense of Competence Scale scores. Measures of video acceptability on a 5-point Likert scale are shown.

	Preintervention Likert scores, mean	Postintervention Likert scores, mean	*P* value
Knowledgeable on care of infant (n=11)	4.09	4.69, mean difference: 0.55 (95% CI −0.27 to 1.36)	.08
Confident in caring for infant (n=10)	4.20	4.80, mean difference: 0.30 (95% CI 0.05 to 1.40)	.02
Generalized Anxiety Disorder-7 (n=12)	4.75	3.33, mean difference: 0.90 (95% CI −3.40 to 0.56)	.07
Parenting Sense of Competence Scale (n=12)	68.5	77.2, mean difference: 3.62 (95% CI −6.12 to 9.79)	.31
Understood material discussed	—^a^	4.8	
Interested in material discussed	—	4.8	
Satisfied with watching videos	—	5	
Satisfied with length of videos	—	4.9	
Videos were well made	—	5	

a Not applicable.

## Discussion

We performed an open-label pilot study evaluating the feasibility and acceptability of videos to increase knowledge and confidence, decrease anxiety, and increase parenting competency of Spanish-speaking fathers of NICU infants. Enrollment feasibility was good (21/27, 78%), but completion of study material could be improved upon (13/21, 62%). Based on high Likert scales on video feedback, the project was accessible and well-accepted. Confidence increased significantly following the intervention, but not anxiety or parenting competence. Reasons for suboptimal study recruitment/retention could include fathers preferring alternative means of communication or poor digital literacy. Ways to improve completion rates of study material include greater incentivization and repeated follow-up (phone calls, emails) [18].

This study has several strengths. The project targets a vulnerable population that is undersupported [2]. We sought an intervention that was portable, flexible, accessible, and did not require NICU presence as fathers visit less than mothers [19,20]. The intervention was low-cost (a couple thousand dollars) and is reproducible for other units, languages, and topics. Fathers gave positive feedback on the videos and suggested other topics as well. This affirmative feedback to improve knowledge is consistent with other data on videos administered in a hospital-based setting that have had significant positive effects on outcomes including knowledge and emotional and behavioral outcomes [21].

The study has several limitations. First, the study sample size was small; significance may have been overestimated and the study was underpowered to detect changes in GAD-7 and PSOC scores or associations between outcomes while controlling for generalized anxiety and clinical characteristics. Second, the study lacked a control group and depended on precomparisons and postcomparisons, making it difficult to establish causality. Second, Latinos are a heterogeneous group and cultural differences may exist that impact study engagement or benefit. Of great importance, multiple factors affect the knowledge, confidence, anxiety, and competence of a father in the NICU; perhaps a single intervention will not influence these factors and a multifactorial approach is necessary. Additionally, the utilized Likert scales were not validated Spanish tools and recall bias may have influenced outcomes. Given this was a feasibility study to elucidate procedures for larger trials, future work will include a control group to allow for external validity. Lastly, fathers self-reported the timing of video watching and number of videos watched; future interventions can confirm videos were watched entirely in a consistent manner.

This preliminary work lends itself to future steps, including improving retention and follow-up through the exploration of barriers to enrollment and retention, as well as a larger, randomized controlled trial powered to assess longer-term changes in outcomes. Due to staffing limitations, we were unable to recruit at all times. Tailoring recruitment during visitation, particularly given that fathers visit less frequently [20], could improve recruitment feasibility and will be included in future work. Finally, the perspective of fathers on their preferred mode of receiving information and support is unknown; we are surveying fathers on their preferred means of communication and content to better target their needs.

## Abbreviations

GAD-7: Generalized Anxiety Disorder-7
NICU: neonatal intensive care unit
PSOC: Parenting Sense of Competence Scale
REDCap: Research Electronic Data Capture
SCN: special care nursery

## References

[1] Stefana A, Padovani EM, Biban P, Lavelli M (2018). Fathers’ experiences with their preterm babies admitted to neonatal intensive care unit: a multi-method study. J Adv Nurs.

[2] Sigurdson K, Morton C, Mitchell B, Profit J (2018). Disparities in NICU quality of care: a qualitative study of family and clinician accounts. J Perinatol.

[3] Cyr-Alves H, Macken L, Hyrkas K (2018). Stress and symptoms of depression in fathers of infants admitted to the NICU. J Obstet Gynecol Neonatal Nurs.

[4] Gutierrez-Galve L, Stein A, Hanington L, Heron J, Ramchandani P (2015). Paternal depression in the postnatal period and child development: mediators and moderators. Pediatrics.

[5] Arockiasamy V, Holsti L, Albersheim S (2008). Fathers’ experiences in the neonatal intensive care unit: a search for control. Pediatrics.

[6] Obregon E, Martin CR, Frantz Iii ID, Patel P, Smith VC (2019). Neonatal Intensive Care Unit discharge preparedness among families with limited English proficiency. J Perinatol.

[7] Ocampo MJ, Tinero JA, Rojas-Ashe EE (2021). Psychosocial interventions and support programs for fathers of NICU infants - a comprehensive review. Early Hum Dev.

[8] Chen YL, Lee TY, Gau ML, Lin KC (2019). The effectiveness of an intervention program for fathers of hospitalized preterm infants on paternal support and attachment 1 month after discharge. J Perinat Neonatal Nurs.

[9] LeDuff LD, Carter BM, Cunningham CA, Braun LA, Gallaher KJ (2021). NICU fathers: improving the quality of paternal support in the NICU. Adv Neonatal Care.

[10] Akbari N, Moradi Z, Sabzi Z, Mehravar F, Fouladinejad M, Asadi L (2021). The effect of narrative writing on fathers’ stress in neonatal intensive care settings. J Matern Fetal Neonatal Med.

[11] Dongre S, Desai S, Nanavati R (2020). Kangaroo father care to reduce paternal stress levels: a prospective observational before-after study. J Neonatal Perinatal Med.

[12] Filippa M, Saliba S, Esseily R, Gratier M, Grandjean D, Kuhn P (2021). Systematic review shows the benefits of involving the fathers of preterm infants in early interventions in neonatal intensive care units. Acta Paediatr.

[13] Hearn G, Clarkson G, Day M (2020). The role of the NICU in Father involvement, beliefs, and confidence: a follow-up qualitative study. Adv Neonatal Care.

[14] Bronfenbrenner U (1994). International Encyclopedia of Education.

[15] (2025). Breastfeeding fast facts. Centers for Disease Control and Prevention.

[16] Teresi JA, Yu X, Stewart AL, Hays RD (2022). Guidelines for designing and evaluating feasibility pilot studies. Med Care.

[17] Gooding K, Phiri M, Peterson I, Parker M, Desmond N (2018). Six dimensions of research trial acceptability: how much, what, when, in what circumstances, to whom and why?. Soc Sci Med.

[18] Booker QS, Austin JD, Balasubramanian BA (2021). Survey strategies to increase participant response rates in primary care research studies. Fam Pract.

[19] Garten L, Maass E, Schmalisch G, Bührer C (2011). O father, where art thou? Parental NICU visiting patterns during the first 28 days of life of very low-birth-weight infants. J Perinat Neonatal Nurs.

[20] Harris LM, Shabanova V, Martinez-Brockman JL (2024). Parent and grandparent neonatal intensive care unit visitation for preterm infants. J Perinatol.

[21] Dahodwala M, Geransar R, Babion J, de Grood J, Sargious P (2018). The impact of the use of video-based educational interventions on patient outcomes in hospital settings: a scoping review. Patient Educ Couns.

